# Mosquitoes

**Published:** 1891-06

**Authors:** 


### MOSQUITOES.

The bill of a mosquito is a complex institution. It has a blunt fork at the head, and is apparently grooved. Working through the groove, and projecting from the angle of the fork, is a lance of perfect form, sharpened with a fine bevel. Beside it the most perfect lance looks like a handsaw. On either side of the lance two saws are arranged, with the points fine and sharp, and the teeth well defined and keen. The backs of these saws play against the lance. When the mosquito lights, with his peculiar hum, it thrusts its keen lance, and then enlarges the aperture with the two saws, which play beside the lance until the forked bill with its capillary arrangement for pumping blood can be inserted. The sawing process is what grates upon the nerves of the victim, and causes him to strike wildly at the sawyer.

